# Supplementary material for: A host-directed oxadiazole compound potentiates antituberculosis treatment via zinc poisoning in human macrophages and in a mouse model of infection
Source: PLoS Biol. 2024 Apr 29;22(4):e3002259. doi: 10.1371/journal.pbio.3002259 (PMC11081512; doi:10.1371/journal.pbio.3002259)
Supplement: S1 Table — (DOCX) [file pbio.3002259.s001.docx]

**S1 Table: Epigenetics compound library used in the study.**

Abbreviation: DNMT: DNA methyltransferase; EZH2: enhancer of zeste homolog 2; HAT: histone acetyltransferase; HDAC: histone deacetylase; PRMT: protein arginine methyltransferases.

| **Number** | **Name** | **Type** | **Cell viability (% compared to DMSO)** | **Spot area (px) per cell (% compared to DMSO)** |
| --- | --- | --- | --- | --- |
| **1** | MC2705 | DNMT Inhibitor | 90.76 | 92.3 |
| **2** | MC2284 | DNMT Inhibitor | 91.88 | 129.54 |
| **3** | MC3688.2HCl | DNMT Inhibitor | 93.81 | 113.84 |
| **4** | MC668.2HCl | DNMT Inhibitor | 94.67 | 112.28 |
| **5** | MC3729.2HCl | DNMT Inhibitor | 99.55 | 99.07 |
| **6** | MC3742 | DNMT Inhibitor | 93.92 | 125.13 |
| **7** | MC3808.2HCl | DNMT Inhibitor | 93.37 | 121.48 |
| **8** | MC3819.2HCl | DNMT Inhibitor | 94.82 | 100.62 |
| **9** | MC3685.2HCl | DNMT Inhibitor | 94.16 | 150.82 |
| **10** | MC3717.HCl | DNMT Inhibitor | 94.28 | 142.25 |
| **11** | MC3696.2HCl | DNMT Inhibitor | 96.22 | 161.91 |
| **12** | MC3695.2HCl | DNMT Inhibitor | 92.72 | 74.53 |
| **13** | MC3743.2HCl | DNMT Inhibitor | 92.56 | 109.7 |
| **14** | MC3974 | DNMT Inhibitor | 88.23 | 84.39 |
| **15** | SGI-1027 | DNMT Inhibito | 95.4 | 92.19 |
| **16** | MC2839 | DNMT Inhibitor | 94.48 | 140.03 |
| **17** | MC2838 | DNMT Inhibitor | 96.54 | 89.27 |
| **18** | MC2835 | DNMT Inhibitor | 94.42 | 105.46 |
| **19** | MC3343 | DNMT Inhibitor | 84.52 | 119.38 |
| **20** | Decitabine | DNMT Inhibitor | 96.91 | 106.91 |
| **21** | MC1742 | Class I, II, IV KDAC Inhibitor | 1.97 | 3.15 |
| **22** | MC2129 | Class I, II, IV KDAC Inhibitor | 67.53 | 103.57 |
| **23** | MS-275 | Class I, II, IV KDAC Inhibitor | 0.35 | 0.79 |
| **24** | MC1510 | Class I, II, IV KDAC Inhibitor | 70.14 | 125.58 |
| **25** | MC1594 | Class I, II, IV KDAC Inhibitor | 67.32 | 126.16 |
| **26** | MC1568 | HDAC6/8 Inhibitor | 95.97 | 91.89 |
| **27** | MC3763 | HDAC6/8 Inhibitor | 101.64 | 105.91 |
| **28** | MC2590 | Class I, II, IV KDAC Inhibitor | 1.33 | 3.54 |
| **29** | MC2664 | Class I, II, IV KDAC Inhibitor | 0.65 | 1.24 |
| **30** | MC2780 | HDAC6 Inhibitor | 15.35 | 74.1 |
| **31** | MC1702 | HDAC8 Inhibitor | 96.01 | 88.5 |
| **32** | MC3108 | HDAC3 Inhibitor | 10.05 | 9.72 |
| **33** | LMK235 | HDAC4/5 Inhibitor | 1.58 | 3.81 |
| **34** | MC2344 | SIRT1/2 inhibitor | 90.83 | 98.54 |
| **35** | MC1776 | SIRT1/2 inhibitor | 101.74 | 88.76 |
| **36** | MC2904 | SIRT2 selective inhibitor | 92.55 | 86.59 |
| **37** | MC3417 | SIRT1/2 inhibitor | 90.67 | 78.77 |
| **38** | MC3465 | SIRT2 selective inhibitor | 89.76 | 44.71 |
| **39** | MC3482 | SIRT5 selective inhibitor | 91.57 | 89.47 |
| **40** | MC2656 | SIRT1/2 inhibitor | 97.04 | 106.22 |
| **41** | MC3422 | SIRT1/2 inhibitor | 77.55 | 90.5 |
| **42** | MC3154 | SIRT6 activator | 90.08 | 97.88 |
| **43** | MDL-800 | SIRT6 activator | 90.92 | 107.72 |
| **44** | MC2562 | SIRT1 activator | 60.5 | 81.86 |
| **45** | MC2791 | SIRT3 activator | 102.35 | 90.53 |
| **46** | MC3215 | SIRT5 activator | 102.78 | 90.72 |
| **47** | MC2055 | PRMT4/Ezh2/HAT inhibitor | 43.97 | 136.12 |
| **48** | MC2089 | PRMT1 inhibitor | 98.63 | 112.39 |
| **49** | MC2298 | PRMT5 inhibitor | 81.97 | 81.54 |
| **50** | MC2908 | EZH2 Inhibitor | 56.38 | 101.84 |
| **51** | Tazemetostat | EZH2 Inhibitor | 86.58 | 97.72 |
| **52** | MC3629 | EZH2 Inhibitor | 95.31 | 112.92 |
| **53** | MC3985 | EZH2 Inhibitor | 99.5 | 89.19 |
| **54** | MC2580 | Histone Demethylase (LSD1) Inhibitor | 92.3 | 100.32 |
| **55** | GSK2879552 | Histone Demethylase (LSD1) Inhibitor | 94.93 | 87.97 |
| **56** | MC4057·3HCl | Histone Demethylase (LSD1) Inhibitor | 0.74 | 1.29 |
| **57** | MC4016·3HCl | Histone Demethylase (LSD1) Inhibitor | 0.38 | 0.52 |
| **58** | GSK690 | Histone Demethylase (LSD1) Inhibitor | 38.73 | 84.25 |
| **59** | IOX-1 | Pan JmjC inhibitor | 79.95 | 135.2 |
| **60** | GSK-J4 | JMJD3 inhibitor - pro-drug | 79.11 | 84.58 |
| **61** | KDM5-C70 (or KDOAM-21) | KDM5 inhibitor | 50.8 | 97.6 |
| **62** | MC3324 | Pan HDM Inhibitor | 94.18 | 52.68 |
| **63** | (+)-JQ1 | Bromodomain (BET) antagonist | 20.18 | 84.25 |
| **64** | C-646 | p300 selective inhibitor | 37.39 | 71.56 |
| **65** | MC4171 | KAT8 selective inhibitor | 93.81 | 97.13 |
| **66** | A-485 | p300 selective inhibitor | 0.31 | 31.71 |
| **67** | Anacardic Acid | p300/PCAF inhibitor | 106.28 | 79.18 |
| **68** | MC4429 | NAT10 inhibitor | 95.45 | 91.58 |
| **69** | MC3353 | DNMT Inhibitor | 68.57 | 203.38 |
| **70** | MC3973 •2HCl | DNMT Inhibitor | 0.67 | 55 |
| **71** | MC3668 ·2HCl | DNMT Inhibitor | 76.58 | 182.28 |
| **72** | MC3863 •2HCl | DNMT Inhibitor | 35.2 | 75.43 |
| **73** | MC4097 | Spindlin antagonist | 117.92 | 142.54 |
| **74** | MC2050‧2HCl | PARP-1 Inhibitor | 63.93 | 93.45 |
| **75** | MC3181 | GST Inhibitor | 84.63 | 60.75 |
| **76** | SirReal2 | SIRT2 Inhibitor | 90.48 | 82.32 |
| **77** | AGK-2 | SIRT2 Inhibitor | 97.8 | 96.64 |
| **78** | EX-527 | SIRT1 Inhibitor | 88.98 | 117.99 |
| **79** | MC2606 | SIRT1 Activator | 53.06 | 107.18 |
| **80** | MC2628 | SIRT1 Activator | 75.66 | 128.34 |
| **81** | MC1621 | HDAC Inhibitor | 69.31 | 186.91 |
| **82** | MC1716 | HDAC Inhibitor | 65.64 | 111.56 |
| **83** | MC1746 | HDAC Inhibitor | 70.23 | 149.05 |
| **84** | MC2062 | HDAC Inhibitor | 94.24 | 169.04 |
| **85** | MC2106 | HDAC Inhibitor | 85.32 | 2.58 |
| **86** | MC2126 | HDAC Inhibitor | 0.86 | 34.61 |
| **87** | MC2141 | SIRT Modulator | 21.75 | 151.95 |
| **88** | MC2183 | SIRT1/2 inhibitor | 91.08 | 110.79 |
| **89** | MC2184 | SIRT1/2 inhibitor | 112.63 | 119.54 |
| **90** | MC2319 | SIRT1/2 inhibitor | 118.87 | 103.43 |
| **91** | MC2336 | SIRT1/2 inhibitor | 94.76 | 105.27 |
| **92** | MC2345 | SIRT1/2 inhibitor | 99.07 | 99.94 |
| **93** | MC2346 | SIRT1/2 inhibitor | 107.29 | 121.98 |
| **94** | MC2358 | SIRT1/2 inhibitor | 21.91 | 106.66 |
| **95** | MC2392 | HDAC Inhibitor | 101.97 | 90.85 |
| **96** | MC2494 | Pan SIRT Inhibitor | 96.71 | 109.61 |
| **97** | MC2905 | SIRT Modulator | 94.09 | 78.54 |
| **98** | MC2907 | SIRT Modulator | 107.08 | 94.7 |
| **99** | MC2933 | SIRT Modulator | 104.1 | 88.13 |
| **100** | MC2935 | SIRT Modulator | 65.33 | 100.71 |
| **101** | MC3163 | SIRT6 activator | 101.33 | 105.56 |
| **102** | MC3179 | SIRT1/2 inhibitor | 111.45 | 93.93 |
| **103** | MC3180 | SIRT1/2 inhibitor | 92.12 | 83.85 |
| **104** | MC3188 | SIRT1/2 inhibitor | 104.08 | 94.27 |
| **105** | MC3196 | SIRT1/2 inhibitor | 102.05 | 85.67 |
| **106** | SIRTINOL | SIRT Modulator | 103.63 | 97 |
| **107** | EML-76 | HAT modulator | 105.84 | 93.91 |
| **108** | MC3595.2HCl | DNMT inhibitor | 97.75 | 132.91 |
| **109** | MC3667.2HCl | EZH2 Inhibitor | 91.18 | 203.98 |
| **110** | MC3742.2HCl | DNMT inhibitor | 75.68 | 142.26 |
| **111** | MC3972 •2HCl | DNMT inhibitor | 34.26 | 4.26 |
| **112** | MC3358 | SIRT Modulator | 1.3 | 63.31 |
| **113** | MC3360 | HAT modulator | 80.68 | 80.51 |
| **114** | MC3226 | SIRT Modulator | 88.69 | 88.61 |
| **115** | MC3321 | Pan Demethylase Inhibitors | 97.39 | 96.96 |
| **116** | MC3004 | HDAC Inhibitors | 92.88 | 112.04 |
| **117** | MC3031 | HDAC Inhibitors | 90.65 | 5.21 |
| **118** | MC3050 | HDAC Inhibitors | 1.4 | 53.43 |
| **119** | MC3073 | Jumonji HDM Inhibitor | 15.14 | 86.18 |
| **120** | MC3076 | HKMT (G9a/GLP) Inhibitor | 84.62 | 129.9 |
| **121** | MC3079 | HDAC Inhibitor | 118.24 | 196.23 |
| **122** | 5-AZA | DNMT Inhibitor | 88.13 | 134.84 |
| **123** | EML-264 | HAT modulator | 118.85 | 120.47 |
| **124** | MC1991 | PRMT/HKMT Inhibitor | 128.47 | 171.49 |
| **125** | MC2288.3HCl | HKMT(G9a/GLP) inhibitor | 76.65 | 223.68 |
| **126** | MC2602 | HDAC Inhibitor | 9.64 | 36.69 |
| **127** | MC2625 | HDAC Inhibitor | 1.53 | 11.42 |
| **128** | MC2652 | Histone Demethylase (LSD1) inhibitor | 88.01 | 142.4 |
| **129** | MC2726 | HDAC Inhibitor | 18.2 | 86.03 |
| **130** | MC2727 | HDAC Inhibitor | 30.7 | 132.06 |
| **131** | MC2887 | HKMT (Ezh2) Inhibitors | 36.98 | 123.89 |
| **132** | MC2840 | DNMT Inhibitor | 77.22 | 212.86 |
| **133** | NBDHEX1 | Glutathione-S-transferase Inhibitor | 1.05 | 64.72 |
| **134** | SPV-106 | HAT modulator | 97.21 | 91.77 |
| **135** | NBDHEX2 | Glutathione-S-transferase Inhibitor | 0.58 | 1.58 |
| **136** | SAHA | HDAC Inhibitor | 1.26 | 1.42 |
| **137** | MC4259 | DNMT Inhibitor | 85.54 | 60.03 |
| **138** | MC4266 | HAT modulator | 0.7 | 2.72 |
| **139** | MC3319 | PRMT/HKMT Inhibitor | 83.47 | 92.55 |
| **140** | MC3401 | HKMT(G9a/GLP) Inhibitor | 76.62 | 75.1 |
| **141** | MC2276 | HDAC Inhibitor | 52.84 | 78.71 |
| **142** | MC2227 | HDAC Inhibitor | 87.27 | 78.47 |
| **143** | MC2282 | SIRT Modulator | 85.29 | 81.5 |
| **144** | MC2177 | SIRT Modulator | 84.14 | 101.33 |
| **145** | MC3544 | SIRT Modulator | 90.64 | 87.92 |
| **146** | MC3946 | SIRT Modulator | 78.23 | 89.88 |
| **147** | MC3677 | SIRT Modulator | 38 | 103.49 |
| **148** | MC3674 | SIRT Modulator | 67.62 | 80.12 |
| **149** | MC3736 | SIRT Modulator | 85.9 | 102.76 |
| **150** | MC4411 | EZH2 Inhibitor | 79.29 | 186.58 |
| **151** | MC4424 | DNMT Inhibitor | 80.41 | 88.18 |
| **152** | MC2418 | DNMT inhibitor | 79.81 | 92.82 |
| **153** | MC2477 | SIRT Modulator | 32.02 | 124.11 |
| **154** | MC2405 | SIRT Modulator | 75.72 | 89.36 |
| **155** | MC2471 | SIRT Modulator | 68.72 | 88.58 |
| **156** | MC3529 | DNMT Inhibitor | 67.79 | 96.72 |
| **157** | MC3512 | DNMT Inhibitor | 56.42 | 109.11 |
