# Supplementary material for: A host-directed oxadiazole compound potentiates antituberculosis treatment via zinc poisoning in human macrophages and in a mouse model of infection
Source: PLoS Biol. 2024 Apr 29;22(4):e3002259. doi: 10.1371/journal.pbio.3002259 (PMC11081512; doi:10.1371/journal.pbio.3002259)
Supplement: S6 Table — (PDF) [file pbio.3002259.s006.pdf]

S6 Table: List and structure of MC3465 analogs.

Compound	Structure	Molecular weight (g/mol)	Spot area (px) per cell (% compared to DMSO)
**MC3465**	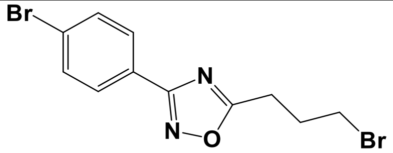	346.022	56.06
**MC3209**	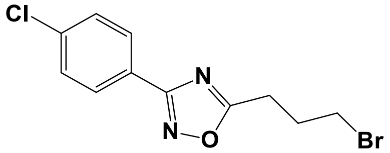	301.568	73.89
**MC3581**	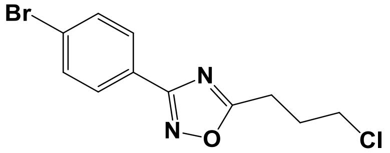	301.568	95.58
**MC3582**	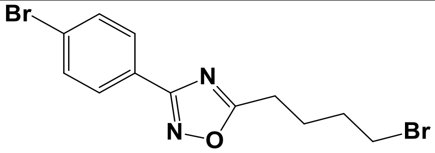	360.049	60.51
**MC3618**	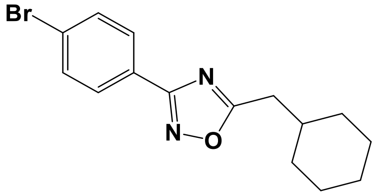	321.218	71.25
**MC3586**	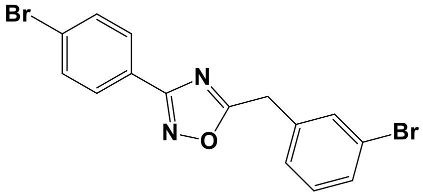	394.066	96.47
**MC3610**	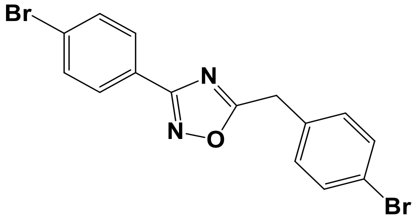	394.066	104.77
**MC3577**	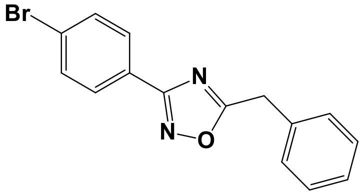	315.170	68.51
**MC3579**	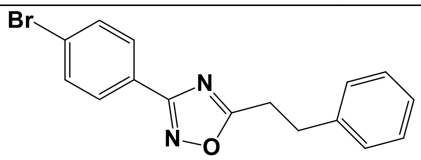	329.197	105.34

**MC3735**	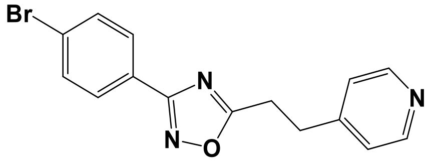	330.185	84.75
**MC3738**	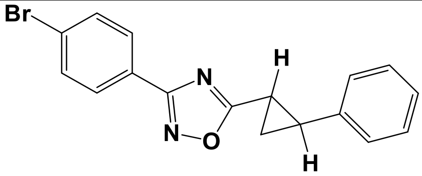	341.208	125.58
**MC3775**	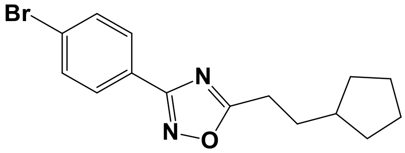	321.218	88.84
**MC3750**	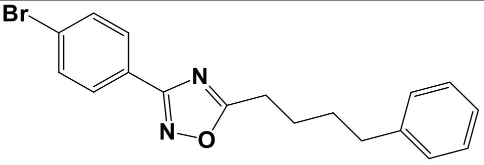	357.251	113.2
**MC3748**	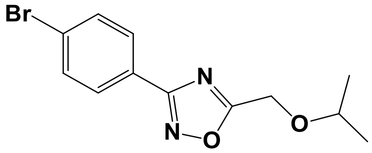	297.152	98.13
**MC3469**	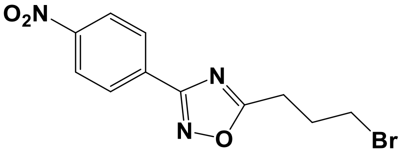	312.123	65.4
**MC3466**	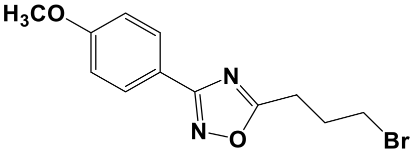	297.152	31.74
**MC3453**	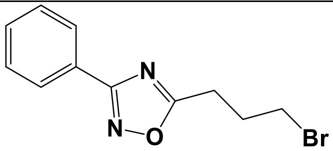	267.126	77.6
**MC3903**	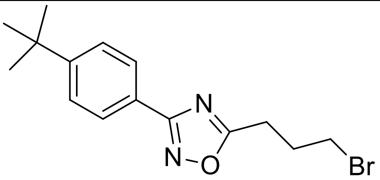	323.234	72.55
**MC3904**	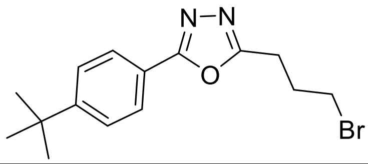	323.234	78.73
**MC3905**	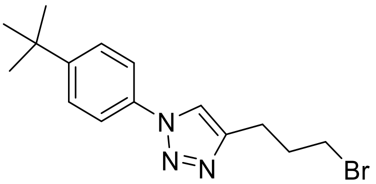	322.250	89.3

**MC3564**	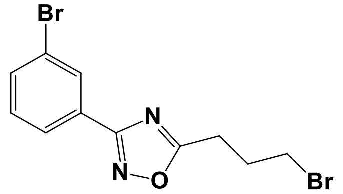	346.022	86.48
**MC3565**	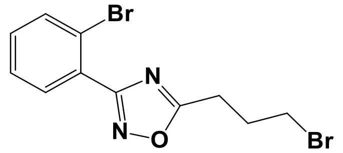	346.022	86.11
**MC3617**	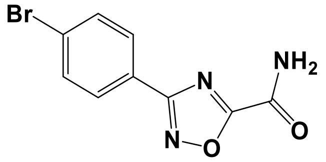	268.070	47.08
**MC3573**	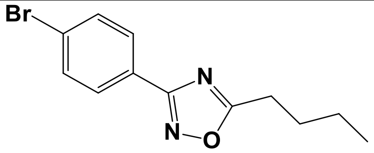	281.153	75.35
**MC3578**	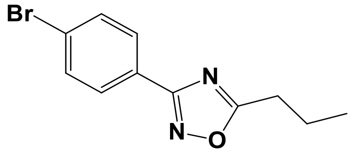	267.126	101.48
**MC3459**	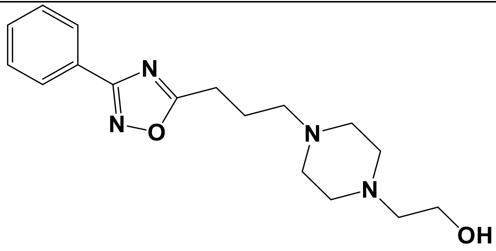	316.405	93.82
**MC3220**	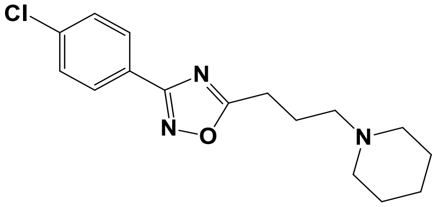	305.806	89.43
**MC3212**	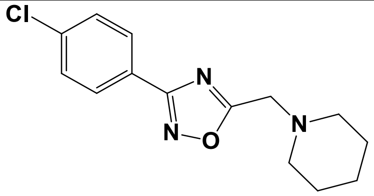	277.752	123.62
**MC4214**	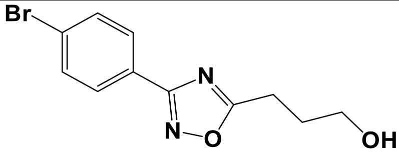	283.125	75.09
**MC4209**	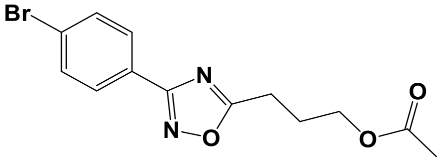	325.162	73.59
