# Supplementary figures and images for: A host-directed oxadiazole compound potentiates antituberculosis treatment via zinc poisoning in human macrophages and in a mouse model of infection
Source: PLoS Biol. 2024 Apr 29;22(4):e3002259. doi: 10.1371/journal.pbio.3002259 (PMC11081512; doi:10.1371/journal.pbio.3002259)

**S4A Fig**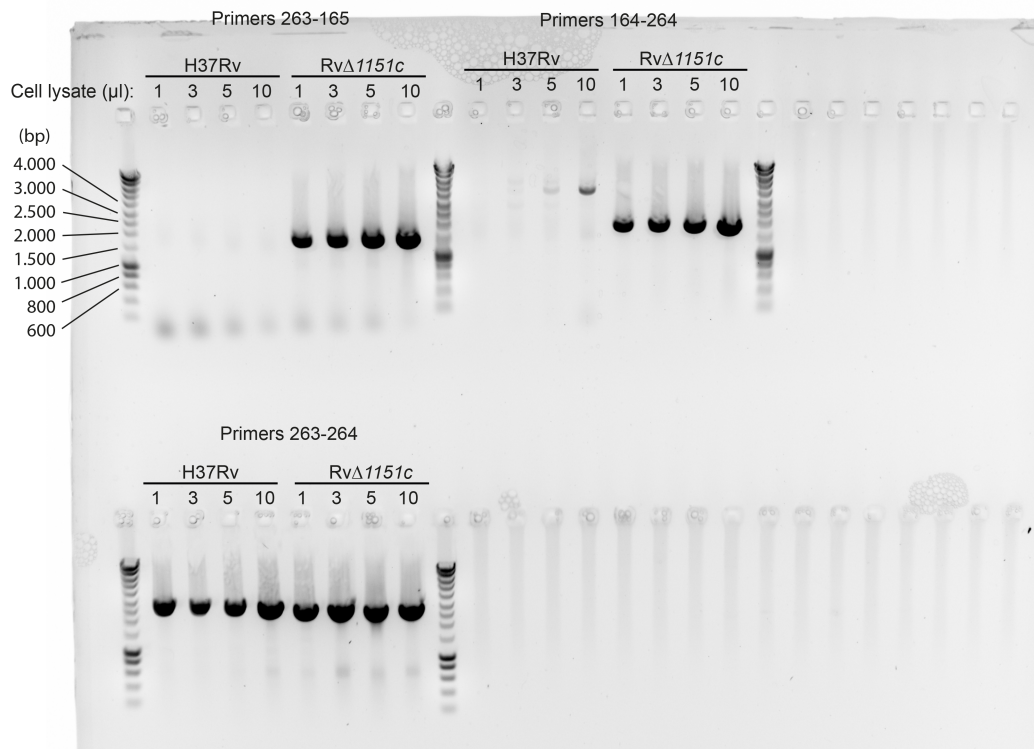**S5C Fig**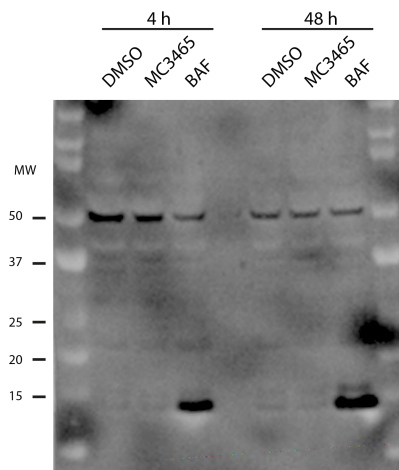

Supplement: S1 Raw Images — (PDF) [file pbio.3002259.s036.pdf]
