# Supplementary material for: A host-directed oxadiazole compound potentiates antituberculosis treatment via zinc poisoning in human macrophages and in a mouse model of infection
Source: PLoS Biol. 2024 Apr 29;22(4):e3002259. doi: 10.1371/journal.pbio.3002259 (PMC11081512; doi:10.1371/journal.pbio.3002259)
Supplement: Attachment — Submitted filename: Maure_response to reviewers.pdf [file pbio.3002259.s037.pdf]

Comments

Reviewer #1: *The manuscript by Maure et al. reports the identification of a novel 1,2,4-oxadiazole-based compound that restricts the growth of Mycobacterium tuberculosis in macrophages, likely through zinc intoxication. In combination with known TB drugs, this compound reduces MTB burden when administered in mice in the acute phase of infection. The manuscript is cogent, the results presented are overall solid and the conclusions sound. However, some of the points below must be addressed while others—although there shouldn't be necessarily addressed—may strengthen the overall robustness of the data presented in the manuscript.*

We thank the reviewer for his positive comments and suggestions, which have enabled us to improve our manuscript significantly.

Major points that should be addressed/discussed:

- *Figure 1: To demonstrate that MC3465 might have specificity towards MTB, the authors show that MC3465 is inactive against Salmonella and Listeria. As accumulation of intracellular concentration of zinc has been shown to be bactericidal against E. coli (Ref. 27), why has the compound not been tested on E. coli?*

We tested MC3465 on *Escherichia coli* infected macrophages. As shown in the figure below, MC3465 treatment had no effect on bacterial load. However, it is important to note that *E. coli* was rapidly eliminated by macrophages. At 24 hours post infection, we detected almost no bacteria. This may explain why MC3465 has no effect while *E. coli* is sensitive to zinc. The macrophage response with the zinc burst was probably already optimal for killing *E. coli*.

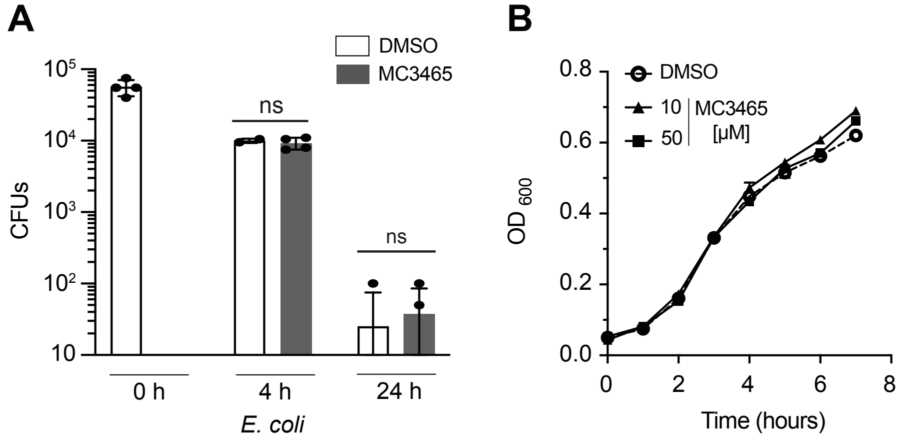

Fig 1. Effect of MC3465 on E. coli infected macrophages

(A) Briefly, *E. coli* (strain HB101) was grown to exponential phase. Bacteria were washed twice in PBS and added to macrophages at a multiplicity of infection (MOI) of 50:1. After 1 h, cells were washed in PBS, and gentamycin (50 µg/ml) was added to kill extracellular bacteria. After an additional 1 h, cells were washed and incubated in fresh complete medium containing gentamycin (5 µg/ml) with DMSO or MC3465 (10 µM). Intracellular bacteria were enumerated at 0, 4 and 24 h post-infection. **(B)** Growth of MTB in liquid culture medium in the presence of MC3465 (10 and 50 µM). One representative experiment (out of two) is shown. Error bars represent the mean ± SD. One-way ANOVA test was used.

- *Figure 3: Why do similar intracellular concentrations of zinc (panel B: - TPEN - MC3465 and + TPEN + MC3465) lead to such differences in CFU counts (panel F)?*

We thank the reviewer for raising this issue. Treatment with the zinc chelator TPEN rapidly and dramatically decreased FluoZin-3 AM fluorescence to near background levels, regardless of whether the cells were treated with MC3465. The cells then became highly susceptible to MTB infection.

With the technique we used, we cannot precisely quantify the amount of intracellular zinc. We can speculate that zinc may be present in the phagosome at levels undetectable by FluoZin-3 AM in the absence of TPEN, and that this may in part limit MTB growth.

- *Figure 4: To add to the lack of correlation between zinc intracellular concentrations and the inhibitors' efficacy, MC3465 is static while MC3466 is cidal, although the former leads to higher accumulation of intracellular zinc than the latter; if this is due to experimental variability, both compounds should be tested in the in same experiment for their effect on the level of intracellular zinc and CFU counts.*

We do indeed have a donor effect. Compounds were more active in some donors than in others. This is not surprising, as the host can affect drug metabolism and efficacy. There are also other

factors to consider, such as the stability of compounds in macrophages. Comparisons between MC3465 and MC3466 were always made using the same cells. In the revised version of our article, we have added MC3465 to the previous Figure 4 (now Figure 5).

- *As MC3465 must have been used as a reference, please indicate where MC3465 is in panel 4A.*

MC3465 has been added to Figure 4A.

- *Figure 6: Does the compound have an effect when administered during the chronic phase of infection? Is the treatment intended for prophylaxis? The rationale of the design of the experiment should be unambiguously stated. In Panel B, the addition of arrows would help guide the naïve eye.*

We did not administer the compound during the chronic phase of infection, but this will be part of our future work. According to our *in vitro* results, MC3466 increases macrophage resistance to MTB, thereby enhancing the efficacy of rifampicin. We therefore wanted to evaluate the efficacy of our compound *in vivo*, focusing on innate immunity. We chose an acute infection model. The rationale of the design of the experiment is now unambiguously stated.
In the Results section, we indicated:
"Our in vitro results showed that our compound enabled human macrophages to fight MTB. We therefore wanted to assess efficacy in an in vivo model where innate immunity plays a key role in controlling infection. We tested our molecule in MTB-infected mice in an acute infection model, alone or in combination with RIF."
In the discussion, we emphasized the importance of studying the efficacy of our compound in different infection models (chronic infection, different mouse strains...).
"MC3466 was tested in an acute infection model. It would be interesting to test its efficacy in a chronic infection model, including various genetic backgrounds with different susceptibility phenotypes. The localization of the compound within the lesions could be studied by taking advantage of the bromine atom contained in the molecule, using an approach similar to that used for BDQ (1)."
In panel B, we added a magnification of each image.

- *Page 16: "improves the healing of lesions". This is an overstatement; the authors did not show that there were any lesions in the first place.*

"improves the healing of lesions" has been replaced by "reduced the number and severity of lesions".

Minor points that should be addressed:

- *"Epigenetic compounds" -reformulate.*

"Epigenetic compounds" has been replaced by “epigenetics-related compounds”

- *In page 5, in the sentence that says: "A 25% and 55% decrease of CFUs", are these numbers correct? For clarity, all variations should be expressed in n fold-reduction.*

The sentence has been changed to read as follows for clarity: "A 2.25- and 4-fold reduction in colony forming units (CFU) was observed after 24 and 96 hours of treatment, respectively, compared to the DMSO control.".

- *Figure 4: MC3465 seems to have less of an effect as compared to panel 1C—is it because the DMSO control grew less? If so, this should be mentioned.*

Depending on the donor, the effects of MC3465 and MC3466 ranged from approximately a 2- to 10-fold reduction in bacterial load after 96 hours of treatment. In some preliminary experiments, we were able to rule out any toxic effect of DMSO. The final concentration of DMSO never exceeded 0.2%. As shown in the S1A-B Fig, DMSO at this concentration had no effect on macrophage viability or MTB growth.

Non-essential, additional work that would strengthen the manuscript:

- *In figure 4, including an inactive analog as a negative control would have been appropriate.*

Thanks for the suggestion. As figure 4 (now figure 5) is already loaded, we have included an additional figure in which Fluozin^{TM} 3-AM fluorescence intensity was quantified after treatment with MC3465 analogs having no effect on MTB growth (S10 Fig).

- *Does the CtpC mutant that cannot efflux zinc have increased sensitivity to MC3465 in macrophage?*

It has been shown recently that PacL1 is essential for maintaining high levels of CtpC and resistance to zinc intoxication (2). We thus generated a triple mutant in which the three operons *pacL1* (Rv3269)-*ctpC*, *pacL2* (Rv1993c)-*ctpG* and *pacL3* (Rv0968)-*ctpV* have been deleted, and which expressed P_{pacL1}-driven CtpC and anhydrotetracycline (Atc)-inducible PacL1. As shown in the revised version of our manuscript, the triple mutant was sensitive to zinc at 200 µM (Fig 4B), and zinc resistance was restored in the presence of Atc (Fig 4B). We infected macrophages with this mutant and treated the cells with DMSO or MC3465 in the presence or absence of Atc. After 96 h of infection, the cells were lysed, and the number of intracellular bacteria was enumerated. In the absence of Atc, MC3465 inhibited the growth of MTB. However, in the presence of Atc, the mutant was able to grow intracellularly at a level similar to bacteria treated with DMSO (Fig 4C).

Overall, these results indicated that MTB is exposed to a high concentration of zinc during treatment with MC3465.

- *Would the authors anticipate that mutants with mutations in ctpC that leads to overexpression of CtpC could be a mechanism of resistance to MC3465? Are there any such mutants found in clinical isolates—that evolved to counter host immunity—that could be tested for their sensitivity to MC3465?*

It is tempting to speculate yes, as we found with our mutant expressing high levels of PacL1 and CtpC. However, to our knowledge, a mutant with mutations in CtpC leading to CtpC overexpression has not been detected yet in clinical isolates.

Reviewer #2: *In the manuscript, the authors have screened a library of 157 epigenetic-modifying molecules to identify novel drugs that restrict Mtb growth in macrophages. The authors propose that the top hit molecules MC3465 and MC3466 are not inhibitors of the SIRT2 histone deacetylase, but rather they induce Zn accumulation inside macrophages to inhibit Mtb replication. Additionally, MC3466 was shown to potentiate the lethality of rifampicin inside macrophages and in an acute mouse model of TB disease.*

We thank the reviewer for his positive feedback and for fair suggestions. Please find our responses below.

Major Concerns:

- *1. While the study shows that MC3465 and MC3466 lead to Zn accumulation in cells which is inhibitory to Mtb, the study falls short of identifying a mechanism for the drug-mediated increase intracellular Zn levels and decrease in metallothionein levels. It would be valuable to know if MC3465 modulates the intracellular levels of other metals such as Cu and Fe? In Mtb-infected macrophages, the Fluozin-3 signal was inhibited by apocynin, an inhibitor of the NADPH phagocyte oxidase, strongly suggesting that the oxidative burst observed during mycobacterial infection leads to the dissociation of zinc from intracellular Zn2+-MT complexes. Does MC3465 induce phox expression and/or its activity?*

We evaluated whether MC3465 modulates intracellular copper levels. MTB infected cells were treated with MC3465. After 4 and 24 h of treatment, cells were labeled with the BioTracker Green copper dye. This is a fluorescent probe that detects Cu+, which is a dominant redox state of

copper in an intracellular reducing environment. We observed no change in staining by MC3465 compared to DMSO (Figure S9). We also attempted to label iron using the BioTracker far-red labile Fe2+ dye. Again, we did not observe any differences with MC3465 treatment. However, the staining was closed to the background, not suitable for publication.

We next investigated whether MC3465 induces reactive oxygen species (ROS) production in both mitochondria and the broader cellular environment. ROS were visualized using two different fluorescent probes: MitoSOX and CellROX. MC3465 did not induce the production of ROS in MTB-infected macrophages at 4 and 24 h post-treatment (S6A-D Fig). As expected, treatment with the antioxidants, glutathione (GSH) and its precursor N-Acetyl-L-cysteine (NAC), did not inhibit the efficacy of MC3465 (S6E Fig).

- *2. Fig. 1a. High throughput screen. A z-value should be provided to measure the robustness of the HTS assay shown in Figure 1A (PMID: 10838414). Also, the data on positive control and negative controls used in the screen should be included. Additionally, in this imaging-based assay, controls where the GFP area/cell are correlated with CFU and MOI would be important to include.*

We provided important information about the quality and robustness of our assay in our revised article. As shown in S1A-B Fig, the concentration of DMSO used in our experiment (0.1%) is not toxic to human cells and does not affect MTB growth. We calculated the Z' factor to assess the quality and reliability of our data (S1C). We found a Z' factor equal to 0.622, indicating a high separation between positive and negative controls. We presented data showing that the GFP area correlated perfectly with CFU (S1D-E), as described previously (3-7). We calculated the z-score for MC3465 (S1F), and it was below -6, showing that MC3465 significantly affected the intracellular MTB growth.

- *3. Fig. 1a. Human MDMs were used. Were these donors tested for latent TB infection (LTBI) by TST and/or IGRA? The authors should mention this in the methods.*

In accordance with French regulations, each blood donation is systematically screened for agents responsible for the following pathologies: AIDS, Hepatitis B, Hepatitis C, Syphilis, and pathologies associated with HTLV. Latent TB infection is not tested. It is now mentioned in the methods.

- *4. P. 5, first full paragraph. It is not clear how molecule-induced cytotoxicity was determined by Hoechst 33342 staining. This dye is cell-permeant and stains both live and dead cells.*

Cells were fixed, washed and labeled with Hoechst 33342. Both live and dead cells can be stained by this dye but only live cells were adherent. Macrophages are cultured in 384-well plates, and

cells remain adherent as long as they are viable. In the presence of a toxic compound, cells undergo necrosis or apoptosis, leading to detachment. In high-throughput screening, nuclear labeling is employed to identify toxic compounds. Treatment with a toxic compound results in a significant reduction in the number of cells in the well compared to untreated wells. In our assay, cells are fixed after 96 hours of treatment with the compounds and labeled with Hoechst 33342. We compared the number of treated cells with the number of cells treated with DMSO (defined as 100%).

- *5. Fig. 1C, 4C: Mtb CFU increase in the presence of MC3465. But the CFU increase is slower than it is with no drug. The authors need to state this clearly. Also, on p. 11 lines 8-13, how can one calculate an IC50 when the bacillary count is actually increasing? Presumably, this was done by setting up proportions to the CFU counts of untreated controls at each time point. In any event, the authors need to state clearly how IC50 was determined.*

We now note that “MC3465 appears to be bacteriostatic or at least significantly reduces the growth of MTB. A 2.25- and 4-fold reduction in colony forming units (CFU) was observed after 24 and 96 hours of treatment, respectively, compared to the DMSO control. (Fig 1C).”
The IC50 was calculated as previously described (5) (now indicated in the manuscript). Briefly, we initially transformed the concentration on the X-axis into log10 concentration. The data were then normalized using the negative control, DMSO (representing 0% inhibition), and the positive control, rifampicin (representing 100% inhibition). Subsequently, we plotted a sigmoidal curve between these two plateaus (0 and 100%), enabling us to determine the IC50 using the DRC by comparing the values obtained with MC3465 or MC3466 with those obtained with DMSO.

- *6. The authors should demonstrate that MC3465 does not directly inhibit SIRT2 activity in vitro as shown in Ref. 18 to rule out this possibility. The previous study (ref. 18) also shows that AGK2 decreases Mtb CFU burden; however, this killing activity was absent in Figure S2A. The authors should address this apparent discrepancy.*

We have presented compelling evidence that MC3465 does not influence the intracellular growth of MTB through the inhibition of SIRT2. Our approach involved pharmacological inhibition of SIRT2, utilizing SIRT2-/- embryonic fibroblasts, and silencing SIRT2 expression in human macrophages through siRNA-mediated gene silencing.
We were also unable to confirm a role for SIRT2 in the control of MTB growth as described by Bhaskar *et al.* (8). In this study, the authors showed that SIRT2 expression is increased in human THP1 cells and in mouse peritoneal macrophages upon MTB infection. However, the effect of SIRT2 inhibition was evaluated in RAW 264.7 macrophages and mouse peritoneal macrophages. They observed that pre-treatment of these cells with AGK2 prior to infection restricted intracellular

MTB growth. As shown in Fig. S3B, pre-treatment of human monocyte-derived macrophages with AGK2 had no effect on MTB growth. The only explanation for these contradictory results lies in the different cell types used in the two studies.
The role of SIRT2 in MTB infection remains unclear, with additional complexities highlighted by Cardoso et al. (9), who found that SIRT2 deletion in the myeloid lineage transiently increased MTB load in the lungs and liver of conditional mice but did not impact long-term infection.
The revised manuscript addresses this apparent discrepancy.

- *7. Figure 2A. The authors state there are 4 groups: DMSO-naive, DMSO-infected, MC3465-naive, MC3465 infected; however, it is not clear what the comparator groups are in Fig. 2a. Please clarify this. Additionally, the cutoff should be at least >1.5 absolute-fold change (log2 FC>0.6) and FDR <0.05, not p-value, so these data should be re-analyzed with appropriate cutoff values. Also, the list of DEGs is not present as a supplementary file, and this list should be added.*

The term naïve refers to uninfected cells. In Fig 2A, only data from MTB-infected macrophages are represented.
We opted for a threshold of FC > 0.5 when selecting differentially expressed genes. The threshold value is arbitrary and depends on the context. In the case of a very strong stimulus that modifies the expression of thousands of genes, it is worth using a high threshold. In our case, despite a relatively small number of differentially expressed genes, we aimed to capture minor changes that could have a significant impact. Therefore, we retained all genes that were statistically differentially expressed with a FC > 0.5.
The list of differentially expressed genes, with corresponding p-values and adjusted p-values, is now provided in supplementary files (S2-5 Tables).

- *8. The authors did not mention why downregulated genes were selected for analysis and what the upregulated genes in the RNA-Seq analysis were. On p. 8, second to last sentence, the term "significantly enriched" is used; the p-values associated with this statement should be provided. Figures 2b and 2c are cytoscape images that do not add meaningful information; these could easily be moved to the supplement.*

The limited number of genes up-regulated by MC3465 hindered the identification of specific GO categories.
The list of these genes is indicated in the supplement (S2-5 tables).
The p-values and the adjusted p-values of the GO terms are now provided in Table 1.
As suggested, we have moved the cytoscape images to the supplement (S7 Fig).

- *9. Page 9, paragraph 1. Ref 27 shows elevated metallothionein (MT) gene expression during Mtb infection is associated with high Zn levels in host cells. Thus, the literature suggests that MT genes are induced at higher concentrations of Zn. The authors therefore need to clarify their claim that lower metallothionein gene expression leads to high labile Zn. Additionally, high concentrations of Zn induce many epigenetic changes and numerous signaling mechanisms in human cells that can indirectly affect host-bacteria interaction and this confounder should at least be discussed (PMID: 26959009).*

Botella *et al*. showed that the expression of several metallothionein-encoding genes was induced upon MTB infection. It has been suggested that these genes were activated in response to the early burst of zinc inside the cells, aiming to decrease free zinc concentration and detoxify the cell. Our observations are not in contradiction with this work. Our results indicate that treatment with MC3465 reduced metallothionein expression, preventing infected cells from efficiently eliminating the released free zinc. Our compound appears to interfere with zinc homeostasis mechanisms.

Metallothionein transcription is regulated by the zinc responsive transcription factor MTF-1 (10). We did not observe any effect of MC3465 treatment on the expression and nuclear translocation of MTF-1. Metallothionein expression is also subject to regulation through epigenetic mechanisms, specifically DNA methylation of the promoter region, which can inhibit their expression (11, 12). It is tempting to speculate that MC3465 might disrupt the epigenetic mechanisms involved in metallothionein transcription. This hypothesis will be evaluated in future work.
This is now discussed in our article

- *10. Figure 3. While Zn is highly accumulated at 4 h, the CFU analysis is done at 96 h. Zn accumulation and CFU should be done at the same timepoints. The authors should provide the Zn levels at later time points (96 h) where there is a bacteriostatic effect to provide direct proof of Zn intoxication.*

In the revised version of our manuscript, we have included the kinetics of FluozinTM 3-AM staining up to 96 h post-treatment, as illustrated in Figure S8C-D. Consistently, we observed an increase in free zinc after 96 h of treatment with MC3465. The difference between the DMSO and MC3465 groups tended to decrease, suggesting that the compound might be less active or degraded by macrophages. It is noteworthy that CFUs were calculated after 96 hours of treatment, reflecting the cumulative impact over four days rather than the instantaneous status of the cells at that specific time. A compound's effect in the initial days can significantly influence MTB growth.

- *11. Figure 3c and 3d. Are the results shown also observed at 96 h? How exactly the phagosomes were counted/determined?*

We detected numerous colocalizations between the FluozinTM 3-AM signal and DsRed-expressing MTBs during the initial time points of MC3465 treatment. However, the difference was no longer statistically significant 96 hours after treatment. As noted above, we cannot rule out the possibility that the MC3465 may be degraded or less active at this time.

The association between dsRed and FluozinTM 3-AM signals was evaluated using the Columbus software. Briefly, bacterial segmentation was performed using the 'Find Spots' building block. The intensity of the FluozinTM 3-AM signal was then calculated within these spots. We have replaced the term "colocalization" with "association" to better reflect our approach.

- *12. Figures 3E and 3F. The concentrations of ZnSo4 and MC3465 needs to be stated. The upregulation of ctpC is marginal and it is unclear if it is statistically significant. Multiple timepoints need to be tested for this experiment. In Ref. 27, the addition of 0.5 mM Zn led to very high ctpC expression, and the authors should offer explanations for the very modest induction reported here. Additionally, either 16S or sigA gene expression is usually used for normalization, rather than the blank which in this case is DMSO.*

The concentrations of $ZnSO_4$ and MC3465 are now indicated in the figure legend.

In the initial version of Fig 3, only the 4-hour time point was displayed for consistency. In the revised version, we now provide the kinetics of Fluozin labeling and *ctpc* expression. Importantly, we confirmed a significant upregulation of *ctpc* gene expression following treatment with both ZnSO4 (consistent with Botella et al.'s findings (13)) and MC3465.

Normalization was performed using the *ftsZ* gene.

- *13. Fig. 6. For mouse infection experiments, the day 0 CFU data would be helpful. Additionally the authors should address the 1000-fold variation in bacterial burden after MC3466-only treatment. For Fig. 6c and p. 23, the authors should clarify who performed the histopathological analysis (was it a trained pathologist?) and whether or is not the scorer was blinded to the identity of the samples.*

We added the day 1 CFU in the revised version of our manuscript.

We have no explanation for the significant heterogeneity observed in the MC3466-treated group (mentioned in the revised manuscript). We ruled out any problems during the mice infection process. Mice were infected simultaneously via the aerosol route with the same bacterial suspension and randomly assigned to each group (control, MC3466, RIF, and MC3466 + RIF). It would be interesting to evaluate in future work whether this heterogeneity decreases with treatment time or infectious dose.

In the Materials and Methods section, we clarified who performed the histopathological analysis and how. We specified, “The analysis was conducted by a trained pathologist from the histopathology core facility at the Institut Pasteur, who was blinded to the treatment group status of individual animals.”

- *14. RNA seq data show reduced expression of genes involved in TNF, type I IFN signaling, and innate immunity alterations. This can reduce inflammation and tissue damage inside the host leading to better treatment outcomes during Mtb infection. Authors should check levels of these cytokines in lungs and also, Zn levels inside mice to support their in vitro data of Zn toxicity to Mtb and disprove other possible reasons.*

While it is technically more challenging to quantify free zinc *in vivo*, particularly in MTB infected mice, we successfully quantified IL-1β level in lung lysates from MTB-infected mice treated for 2 weeks with vehicle, MC3466, rifampicin (RIF), or the combination of RIF + MC3466. As shown in Figure 7D, treatment with MC3466 resulted in a reduction in IL-1β levels, though to a lesser extent than observed with RIF alone. The combination of RIF and MC3466 did not further decrease IL-1β levels compared to RIF. This reduction is likely associated with a decrease in bacterial load rather than an anti-inflammatory activity of MC3466.

- *15. Statistical analysis and scientific rigor. Please show the number of biological and technical replicates; this information is missing in many experiments. For Fig 1 and Fig. 3 it is impossible to tell which panel used T-test and which used ANOVA. The data figures would be strengthened by showing all data points in addition to the SD. In Fig. 4c, 5a, and 5b it is unclear which groups are being compared for the p values shown.*

This has been corrected in the revised version of our manuscript.

Minor Concerns:

- *16. P. 3, lines 15-16. MDR-TB is now readily treatable with the BPaL regimen. Please see Conradie et al NEJM 2022 PMID 36053506.*

The sentence “Curing MDR-TB is more difficult and requires treatments of up to 2 years with more toxic drugs, often with limited success.” has been replaced by “Curing MDR-TB is more difficult, despite recent advances (Conradie, 2022)”.

- *17. Fig. S3b and P. 8, line 4: The figure shows that LC3B puncta are lower in MC3465-treated cells at the early time point (p< 0.001), while the text states that there was a minimal difference. Also, there is a discrepancy between the figure (which says the early time point is 4 h) and the text (which says that the early time point is 24 h). Additionally, a*

Western blot must be done to see unchanged LC3 I and II levels to detect alterations in autophagy at 4h and 96 h.

Autophagy was assessed at 4 and 48 hours after MC3465 treatment. Microscopic examination revealed a decrease in the number of LC3B puncta per cell at 4 hours (DMSO: mean 5.172 +/- SD 4.216, MC3465: 3.310 +/- 3.363), suggesting a potential inhibition of autophagy by MC3465. However, Western blot analysis (now shown in Figure S5C) did not confirm these results, as no difference was observed between control and MC3465-treated cells.

To further investigate the impact on autophagy, we treated MTB-infected cells with MC3465 alongside well-characterized autophagy inhibitors. There were no observable effects on the ability of MC3465 to restrict MTB growth. These findings lead us to conclude that MC3465 does not limit mycobacterial growth by inducing autophagy.

The text has been revised accordingly.

- *18. P. 12 line 15-17. Neither the figure nor the text state what the Bliss scores are. Please provide the actual score. The methodology should be properly written so that others can repeat the experiment if required. The color code does not match or is not clear to me.*

The manuscript now includes a clear description of the Bliss Independence model, elucidating how the Excess over Bliss score is calculated for evaluating drug interactions. There was an error in the scale of the heat map. Negative signs were absent. This error has been corrected.

- *19. P. 13, lines 5-8. It is noted that there were no adverse effects on AST and ALT, but as a drug that modulates metals in cells, one would be concerned about marrow toxicity and the levels of RBC, WBC, platelets, and hemoglobin. Were these tested?*

A drug can potentially have adverse effects on various physiological systems. Therefore, evaluating its toxicity in mice requires the examination of numerous parameters. In this study, we monitored for signs of distress, abnormal behavior, or changes in body weight. Additionally, we measured liver enzymes (ALT, AST) as indicators of liver function.

We acknowledge that these parameters alone may not be sufficient to conclusively determine the compound's toxicity. We did not assess bone marrow, cardiac, or reproductive toxicity, nor did we quantify levels of RBCs, WBCs, platelets, and hemoglobin. While recognizing the need for further studies, we believe these aspects extend beyond the scope of our current manuscript. These additional investigations would represent the initial steps in the preclinical evaluation of our compound. In the revised version of our article, we have replaced the term 'non-toxic' with 'well-tolerated' to better reflect our findings.

- *20. Methods are not properly written in the manuscript. It is unclear what macrophages were used and what were the concentrations of different molecules in many experiments.*

Unless otherwise noted in the figure legend, experiments were performed on human monocyte-derived macrophages. The concentration of the different molecules is now given in the legend of each figure.

- *21. References are duplicated. Please correct these errors and correct others, if any.*

These errors have been corrected in the new version of our article.

- *22. Some figure labels are incorrect in the text. Authors should correct these errors.*

Thank you for pointing out these errors, which have been corrected in the revised version of our manuscript.

- *23. For growth-based experiments, CFU should be measured and not OD.*

The growth of MTB in liquid culture medium in the presence of MC3465 or MC3466 was monitored by measuring the optical density (OD) or enumerating colony-forming units (CFUs) at various time points. CFU results are now presented in Supplementary Figures S2C and S10A.

- *24. Authors should show the data (PCR or qRT-PCR) supporting that rv1151c gene is deleted in the KO generated in Mtb*.

PCRs demonstrating Rv1151c gene knockout are now presented in S4A Fig.

- *25. The authors should mention why an MOI of 50 was used for S. typhimurium and L. monocytogenes in Figure 1E and MOI= 0.5 for Mtb. Additionally, 0h and 96 h CFU were absent in the experiment.*

The methods for infecting cells with slow-growing and fast-growing bacteria generally differ. Another crucial factor to consider is the bacteria's ability to survive and replicate within macrophages (as mentioned in our response to the other reviewer regarding *E. coli)*. Our infection protocol aligns with commonly used practices in the literature, such as those for *L. monocytogenes* (14, 15) or *S*. Typhimurium (16-18). We have indicated this point in the Materials and Methods of our article.

For fast-growing bacteria, higher Multiplicity of Infection (MOI) values (ranging from 10 to 100:1) and shorter infection times (30 minutes to 1 hour) are applied, followed by a 1-hour incubation with gentamicin to eliminate extracellular bacteria.

MTB employs various strategies for multiplication within macrophages. Infecting these cells with a high MOI of MTB often results in increased macrophage mortality. Given MTB's significantly longer doubling time (20 minutes for *Salmonella*, 45-60 minutes for *Listeria*, and around 24 h for MTB), our experimental kinetics extend up to 96 h.

- *26. Why did the time point change to 48h in Figure S6A? Authors should use the same time point for comparison in the study.*

RAW 264.7 is a macrophage cell line established from an Abelson-Murine Leukemia Virus-induced tumor. In contrast to human monocyte-derived macrophages, RAW 264.7 cells exhibit rapid proliferation (with an average doubling time of approximately 15 hours) and undergo cell death upon reaching confluence. Consequently, treating these cells for 96 hours without splitting them was not feasible.

- *27. Data point outside the x-axis in 4F. How was this data plotted?*

Thank you for reporting this issue. The graphics were created in Prism and transferred to Adobe Illustrator for figure creation. An error may have occurred during this process.

References

1. Greenwood DJ, Dos Santos MS, Huang S, Russell MRG, Collinson LM, MacRae JI, et al. Subcellular antibiotic visualization reveals a dynamic drug reservoir in infected macrophages. Science. 2019;364(6447):1279-82.
2. Boudehen YM, Faucher M, Marechal X, Miras R, Rech J, Rombouts Y, et al. Mycobacterial resistance to zinc poisoning requires assembly of P-ATPase-containing membrane metal efflux platforms. Nat Commun. 2022;13(1):4731.
3. Ahmed S, Manning A, Flint L, Awasthi D, Ovechkina Y, Parish T. Identification of Novel Chemical Scaffolds that Inhibit the Growth of Mycobacterium tuberculosis in Macrophages. Front Pharmacol. 2021;12:790583.
4. Brodin P, Poquet Y, Levillain F, Peguillet I, Larrouy-Maumus G, Gilleron M, et al. High content phenotypic cell-based visual screen identifies Mycobacterium tuberculosis acyltrehalose-containing glycolipids involved in phagosome remodeling. PLoS Pathog. 2010;6(9):e1001100.
5. Christophe T, Jackson M, Jeon HK, Fenistein D, Contreras-Dominguez M, Kim J, et al. High content screening identifies decaprenyl-phosphoribose 2' epimerase as a target for intracellular antimycobacterial inhibitors. PLoS Pathog. 2009;5(10):e1000645.
6. Han HW, Seo HH, Jo HY, Han HJ, Falcao VCA, Delorme V, et al. Drug Discovery Platform Targeting M. tuberculosis with Human Embryonic Stem Cell-Derived Macrophages. Stem Cell Reports. 2019;13(6):980-91.
7. Manning AJ, Ovechkina Y, McGillivray A, Flint L, Roberts DM, Parish T. A high content microscopy assay to determine drug activity against intracellular Mycobacterium tuberculosis. Methods. 2017;127:3-11.
8. Bhaskar A, Kumar S, Khan MZ, Singh A, Dwivedi VP, Nandicoori VK. Host sirtuin 2 as an immunotherapeutic target against tuberculosis. Elife. 2020;9.
9. Cardoso F, Castro F, Moreira-Teixeira L, Sousa J, Torrado E, Silvestre R, et al. Myeloid Sirtuin 2 Expression Does Not Impact Long-Term Mycobacterium tuberculosis Control. PLoS One. 2015;10(7):e0131904.
10. Kimura T, Kambe T. The Functions of Metallothionein and ZIP and ZnT Transporters: An Overview and Perspective. Int J Mol Sci. 2016;17(3):336.
11. Ghoshal K, Majumder S, Li Z, Dong X, Jacob ST. Suppression of metallothionein gene expression in a rat hepatoma because of promoter-specific DNA methylation. J Biol Chem. 2000;275(1):539-47.
12. Majumder S, Ghoshal K, Li Z, Bo Y, Jacob ST. Silencing of metallothionein-I gene in mouse lymphosarcoma cells by methylation. Oncogene. 1999;18(46):6287-95.

13. Botella H, Peyron P, Levillain F, Poincloux R, Poquet Y, Brandli I, et al. Mycobacterial p(1)-type ATPases mediate resistance to zinc poisoning in human macrophages. Cell Host Microbe. 2011;10(3):248-59.
14. Lebreton A, Lakisic G, Job V, Fritsch L, Tham TN, Camejo A, et al. A bacterial protein targets the BAHD1 chromatin complex to stimulate type III interferon response. Science. 2011;331(6022):1319-21.
15. Pereira JM, Chevalier C, Chaze T, Gianetto Q, Impens F, Matondo M, et al. Infection Reveals a Modification of SIRT2 Critical for Chromatin Association. Cell Rep. 2018;23(4):1124-37.
16. Gram AM, Wright JA, Pickering RJ, Lam NL, Booty LM, Webster SJ, et al. Salmonella Flagellin Activates NAIP/NLRC4 and Canonical NLRP3 Inflammasomes in Human Macrophages. J Immunol. 2021;206(3):631-40.
17. Selkrig J, Li N, Hausmann A, Mangan MSJ, Zietek M, Mateus A, et al. Spatiotemporal proteomics uncovers cathepsin-dependent macrophage cell death during Salmonella infection. Nat Microbiol. 2020;5(9):1119-33.
18. Xiong L, Wang S, Dean JW, Oliff KN, Jobin C, Curtiss R, 3rd, et al. Group 3 innate lymphoid cell pyroptosis represents a host defence mechanism against Salmonella infection. Nat Microbiol. 2022;7(7):1087-99.
